# Metformin induces ZFP36 by mTORC1 inhibition in cervical cancer-derived cell lines

**DOI:** 10.1186/s12885-024-12555-5

**Published:** 2024-07-18

**Authors:** Karen Griselda De la Cruz-López, Eduardo Alvarado-Ortiz, Heriberto A. Valencia-González, Fredy Omar Beltrán-Anaya, José María Zamora-Fuentes, Alfredo Hidalgo-Miranda, Elizabeth Ortiz-Sánchez, Jesús Espinal-Enríquez, Alejandro García-Carrancá

**Affiliations:** 1 Programa de Doctorado en Ciencias Biomédicas, Instituto de Investigaciones Biomédicas, Universidad Nacional Autónoma de México, Mexico City, Mexico; 2 Departamento de Bioquímica, Facultad de Medicina, Universidad Nacional Autónoma de México, Mexico City, Mexico; 3 Laboratorio de Genómica de Cáncer, Instituto Nacional de Medicina Genómica, Mexico City, Mexico; 4 Laboratorio de Diagnóstico e Investigación en Salud, Facultad de Ciencias Químico-Biológicas, Universidad Autónoma de Guerrero, Chilpancingo de los Bravo, Gro Mexico; 5 Laboratorio de Oncología Teórica, Instituto Nacional de Medicina Genómica, Mexico City, Mexico; 6 Centro de Ciencias de La Complejidad, Universidad Nacional Autónoma de México, Mexico City, Mexico; 7 Laboratorio de Células Troncales y Desarrollo Terapéutico Antineoplásico, Subdirección de Investigación Básica, Instituto Nacional de Cancerología, Secretaría de Salud, Mexico City, Mexico; 8 Laboratorio de Virus y Cáncer, Unidad de Investigación Biomédica en Cáncer. Instituto de Investigaciones Biomédicas, Universidad Nacional Autónoma de México & Instituto Nacional de Cancerología., Av. San Fernando No. 22 Colonia Sección XVI, Tlalpan, Mexico City, 14080 Mexico

**Keywords:** Metformin, ZFP36, AMPK-independent, mTORC1

## Abstract

**Background:**

Metformin, a widely prescribed antidiabetic drug, has shown several promising effects for cancer treatment. These effects have been shown to be mediated by dual modulation of the AMPK-mTORC1 axis, where AMPK acts upstream of mTORC1 to decrease its activity. Nevertheless, alternative pathways have been recently discovered suggesting that metformin can act through of different targets regulation.

**Methods:**

We performed a transcriptome screening analysis using HeLa xenograft tumors generated in NOD-SCID mice treated with or without metformin to examine genes regulated by metformin. Western Blot analysis, Immunohistochemical staining, and RT-qPCR were used to confirm alterations in gene expression. The TNMplot and GEPIA2 platform were used for in silico analysis of genes found up-regulated by metformin, in cervical cancer patients. We performed an AMPK knock-down using AMPK-targeted siRNAs and mTOR inhibition with rapamycin to investigate the molecular mechanisms underlying the effect of metformin in cervical cancer cell lines.

**Results:**

We shown that metformin decreases tumor growth and increased the expression of a group of antitumoral genes involved in DNA-binding transcription activator activity, hormonal response, and Dcp1-Dcp2 mRNA-decapping complex. We demonstrated that ZFP36 could act as a new molecular target increased by metformin. mTORC1 inhibition using rapamycin induces ZFP36 expression, which could suggest that metformin increases ZFP36 expression and requires mTORC1 inhibition for such effect. Surprisingly, in HeLa cells AMPK inhibition did not affect ZFP36 expression, suggesting that additional signal transducers related to suppressing mTORC1 activity, could be involved.

**Conclusions:**

These results highlight the importance of ZFP36 activation in response to metformin treatment involving mTORC1 inhibition.

**Graphical Abstract:**

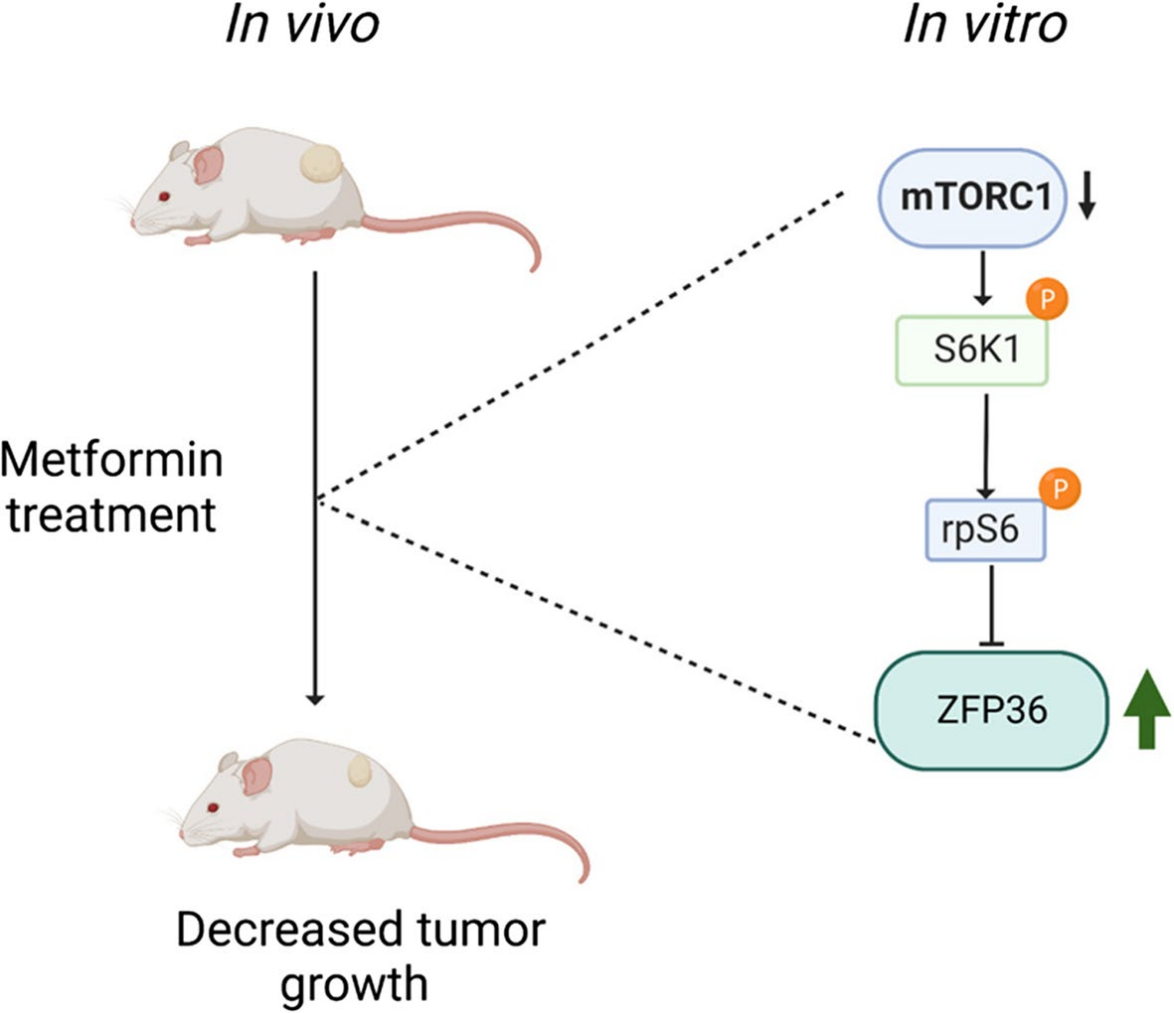

**Supplementary Information:**

The online version contains supplementary material available at 10.1186/s12885-024-12555-5.

## Introduction

Metformin (1,1-dimethylbiguanide hydrochloride) is the first-line oral drug used for type 2 diabetes. Accumulative evidence indicate that this oral drug possesses several potentials and attractive antitumoral effects [1, 2]. Epidemiological studies suggest that metformin shows promising effects on gynecologic malignancies, mainly a significant decreased risk was identified in cervical cancer (CC) (RR: 0.60, 95% CI: 0.43–0.83, and *p* = 0.002) [3, 4]. CC is the fourth leading cause of cancer-related death among women worldwide, from which a 30% present diabetes at the time of diagnosis [5–7]. Interestingly, female patients with type 2 diabetes taking metformin showed a lower cervical cancer incidence, especially those with longer treatment regimen [4]. In addition, CC patients with cumulative metformin use after diagnosis have a decreased mortality rate, mainly of squamous cell carcinomas [8].

At cellular level, metformin partially and reversibly inhibits the mitochondrial complex I (NADH dehydrogenase), leading to electron transport chain (ETC) inhibition lowering ATP synthesis and increasing AMP levels activating the AMP-activated protein kinase (AMPK) [9]. AMPK acts as a major cellular energy sensor that indirectly inhibits the mechanistic target of rapamycin complex 1 (mTORC1) [10]. mTORC1 regulates protein synthesis and ultimately cell growth and proliferation by phosphorylating two main targets, the ribosomal protein S6 kinase 1 (S6K1) and the eukaryotic initiation factor 4E-binding protein (4E-BP).

S6K1 activation by mTORC1 leads to increased mRNA biogenesis and cap-dependent translation [11]. S6K1 consists of two isoforms: 70-kDa cytoplasmic isoform (p70S6K) and 85-kDa nuclear isoform (p85S6K), both isoforms have multiple substrates, including the ribosomal protein S6 (rpS6), whose phosphorylation is tightly coupled to mTORC1 activity [12]. RpS6 is a key constituent of the ribosomes 40S subunit controlling protein synthesis, ribosomal biogenesis, and cell size [13]. Additionally, rpS6 is also phosphorylated by the p90 ribosomal protein S6 kinase (P90S6K, also known as RSK), via MAPK/Erk/P90S6K in response to innumerable mitogenic signals, such as insulin and epidermal growth factor (EGF) [14].

In several cancers, such as breast or endometrial carcinoma, metformin has produced an inhibition of cell growth by decreasing mTORC1 activity [15, 16]. The antitumoral effects of metformin are known to rely on mTORC1 axis inhibition via the activation of AMPK. However, it has been recently suggested that activation of AMPK might be dispensable for this inhibition [17–19]. The existence of additional pathways leading to mTORC1 inhibition, as well as the metformin pleiotropic effects demonstrated in some cancers, suggests that metformin might act through alternative pathways to AMPK, opening new approaches underling the antitumoral metformin effects.

The main goal of this study was to deepen into the molecular mechanism regulated by metformin using xenograft-derived tumors and cervical cancer-derived cell lines. Therefore, a transcriptome screening analysis using xenografted tumors formed in mice treated with or without metformin was performed, to identify transcriptional metformin targets, as well as important signaling pathways. We found that metformin regulates genes involved in DNA-binding transcription activator activity, hormonal response, and Dcp1-Dcp2 mRNA-decapping complex. We discovered that metformin induces ZFP36 expression in vivo and in vitro. Our results support the idea that ZFP36 induction occurs through mTORC1 inhibition, providing novel mechanisms involved in the metformin effects on tumor growth suppression and inhibition of cancer-derived cell lines. Our work extends the knowledge about mechanisms involved in cervical cancer inhibition and ZFP36 regulation by metformin.

## Materials and methods

### Cell culture

Primary Human Uterine Fibroblast (HUF) (PCS-460–010) and human cervical cancer-derived cell lines HeLa (RRID: CVCL_0030) and CaSki (RRID: CVCL_1100) were obtained from ATCC (Manassas, VA, USA). Human keratinocyte cell line HaCaT was kindly provided by Dr. Sergio Manuel Encarnación Guevara (CCG-UNAM). HeLa cells were cultured in Dulbecco’s Modified Eagle Medium–high glucose (DMEM, Gibco 11,965–084). CaSki and HaCaT cells were cultured in Roswell Park Memorial Institute (RPMI) 1640 medium. Both media were supplemented with 10% of fetal bovine serum (FBS) (Gibco-10500056) and 100 U/mL–100 µg/mL of penicillin–streptomycin (Invitrogen, USA). HUF were cultured in Dulbecco’s Modified Eagle F12 (DMEM F12) supplemented with 5% of FBS. Cell cultures were maintained in a humidified environment containing 5% CO_2_ at 37ºC. All experiments were performed using mycoplasma-free cells.

### Metformin treatments in vitro and viability assay

Metformin (1,1-dimethylbiguanide hydrochloride, Sigma-Aldrich, USA, D150959) was freshly diluted in either DMEM or RPMI medium at 1 M concentration for all experiments. Cells were seeded (450,000–600,000 cells) in 60 mm plates and incubated with medium containing 10% FBS. After 24 h, medium was replaced with fresh medium, and then treated with different concentrations of metformin at 5 mM, 10 mM, and 20 mM for 24 h. For mTORC1 inhibition, rapamycin (Sigma-Aldrich, USA, SML2282) was prepared in 96% ethanol. Cells were seeded (250,000 cells) in 30 mm plates and treated with rapamycin at 50 nM or with 96% ethanol as control for 24 h.

For viability assay, cells were seeded (50,000 cells/well) in 96-well plates and incubated with medium containing 10% FBS for 24 h. Then, medium was replaced with fresh medium containing metformin at 5 mM, 10 mM, and 20 mM and incubated for another 24 h. Subsequently, cell viability was determined using MTT (3-(4,5-Dimethylthiazol-2-yl)-2,5-Diphenyltetrazolium bromide) as previously described [20]. Absorbance was measured at 570 nm using an Epoch microplate spectrophotometer (Biotek Instrument, USA).

### Western blot

Cell and tumor tissues lysates were obtained using a lysis buffer composed of 50 mM Tris, pH 7.6, 150 mM NaCl, 5 mM EDTA, 1% Triton, and a complete tablet protease Inhibitor Cocktail (Sigma-Aldrich, USA, S8830) in 100 ml of buffer and 1X phosphatase inhibitor cocktail (Cell Signaling Technology, Beverly MA, USA, 5870S). Protein concentrations were measured using the Pierce BCA Protein Assay Kit (Thermo Fisher Scientific, USA, 23,225) according to the manufacturer´s instructions. Equal amounts of total protein (15 μg) were separated by 10% sodium dodecyl sulfate–polyacrylamide gel electrophoresis (SDS-PAGE) and transferred onto nitrocellulose membranes. Membranes were blocked with 5% Bovine Serum Albumin in TBS-Tween (0.05% Tween-20 in Tris-buffered solution) for one hour and incubated overnight at 4ºC with the appropriate primary antibody dilution (ranges from 1:1000 to 1:2000). Membranes were washed using TBS-Tween and incubated with the appropriate HRP-conjugated secondary antibody. Immunodetection was performed with an enhanced chemiluminescence reaction using the Immobilon ECL Ultra Western HRP substrate (Merck Millipore, USA) according to the manufacturer´s instructions and visualized using a C-DiGit chemiluminescence Western Blot scanner (LI-COR Biosciences, Lincoln, Nebraska USA, 3600). The following antibodies were used: anti-p-AMPK (Thr172) (2535S), anti-AMPK (2532S), anti-p-rpS6 (Ser235/236) (2211), anti-rpS6 (2317), anti-p-S6K1 (Thr389) (D5U10) (97,596), anti-S6K1 (49D7) (2708) (Cell Signaling Technology, Beverly MA, USA). Anti-ZFP36 (T5327) (Sigma-Aldrich). Anti-p-Erk1/2 (Tyr204) (sc7383), anti-Erk2 (sc-154), anti-glyceraldehyde-3-phosphate dehydrogenase (GAPDH) (L-18) (sc-48167), anti-goat (sc-2020), anti-mouse (sc-2005), and anti-rabbit (sc-2013) (Santa Cruz Biotechnology Inc, CA, USA).

### AMPK knockdown using siRNA

HeLa cells were transfected with siRNA targeting the α1 (PRKAA1) and α2 (PRKAA2) isoforms of AMPK with the SMARTpool siGENOME platform (Dharmacon). All transfections were done using Lipofectamine 3000 (Invitrogen, Waltham, MA, USA) in Opti-MEM (Gibco, USA) according to the manufacturer’s instructions. For each transfection, we used a 100 nM final concentration of either siGENOME non-Targeting siRNA #1 or 50 nM SMARTpool siGENOME PRKAA1 and 50 nM SMARTpool siGENOME PRKAA2 siRNA, referred hereinafter as Scramble and AMPK siRNAs, respectively. Cells were washed during 24 h after transfection to eliminate any trace of transfection reagents and medium was added. Appropriate volumes of medium containing freshly diluted metformin were added for a final concentration of 20 mM and cells were incubated for another 24 h.

### Tumor xenografts and oral metformin treatments

Non-Obese Diabetic-Severe Combined Immunodeficiency (NOD-SCID) female mice aged 4–6 weeks were obtained from *Unidad de Modelos Biológicos, Instituto de Investigaciones Biomédicas, UNAM*. All experimental procedures were generated according to ethical regulations, methodologies and protocols approved by the corresponding Research and Bioethical Committees of Instituto Nacional de Cancerología Mexico City, MEXICO (Approval No: (019/039/IBI) (CEI/1252/18)).

All mice were housed and bred in Specific-Pathogen-Free (SPF) conditions at 22–25 ºC, 40–60% humidity, and 12 h/12 h light/dark cycles. A HeLa cell suspension containing 1.5X10^6^ cells in a volume of 200 μl was injected subcutaneously into the left flank of each mouse. Tumor xenografts were allowed to develop to an average size of 60 to 80 mm^3^ before metformin treatment. Tumor growth was measured using the Attia-Weiss formula: “Tumor volume = (0.4) (a) (b^2)”, where “a” is the largest diameter and “b” is the smallest diameter of each tumor [21]. Diameters were measured using digital Vernier Calipers.

Once tumors were established, mice were treated daily with 525 mg/kg/day metformin via gastric gavage for 24 days. The dose was established according to ranges used in similar experiments that have not shown any toxic effect [22–24]. Metformin freshly diluted in water was orally administered daily via gavage (curved, 20X1.5’’). Tumor volume was measured every third day. After completing metformin treatment, mice were euthanized in a compressed CO_2_ chamber, and tumors were extracted, measured, weighed, and photographed. Part of the tumor was frozen in liquid nitrogen for RNA and protein extraction while the rest was fixed in buffered 4% paraformaldehyde for immunohistochemistry assays.

### Immunohistochemical (IHC) staining

Sections from tumor xenografts were stained and IHC analysis was performed as previously reported [25]. Briefly, 5 μm thick paraffin-embedded tumor sections were deparaffinized, rehydrated, and stained with hematoxylin and eosin (H&E). Slides were dehydrated with ethanol, then epitope retrieval was done using ImmunoRetriever Citrate Solution (Bio SB, USA) for 12 min. Non-specific binding was blocked using 10% BSA for 30 min and slides were incubated overnight with primary antibodies against Ki67 (Abcam Cambridge, UK, ab16667) and p-rpS6 (Ser 235/236) (Cell Signaling Technology, Beverly MA, USA, 2211). The Mouse/Rabbit PolyDetector horseradish HRP/DAB Detection System was used according to the manufacturer’s recommendations (Bio SB, USA, BSB 0205). The photographs were taken using Axio Vert.A1 inverted microscope (Carl Zeiss).

### RNA isolation

Total RNA was extracted using the RNeasy Mini Kit (Qiagen, Germany) according to the manufacturer’s recommendations. Subsequently, RNA was quantified by Nanodrop spectrophotometer (Thermo Fisher Scientific, USA) and stored at -80ºC until further processing.

### mRNA Microarrays and gene expression analysis

RNA quality obtained from the tumor tissues was evaluated by capillary electrophoresis (Agilent 2100 Bioanalyzer; Agilent Technologies). Samples with an RNA Integrity Number (RIN) greater than six (6.0) were further processed for microarray studies. From each experimental condition, 100 ng of RNA were evaluated using the GeneChip Human Transcriptome Array (HTA) 2.0 (Affymetrix, Santa Clara, CA, USA). Complementary DNA (cDNA) synthesis, amplification, and labeling were done using the WT plus reagent kit and WT Labeling kit for fresh samples (Affymetrix, Santa Clara, CA, USA), and then cDNA was hybridized on the arrays. Arrays were washed (Fluidics Station 450), stained, and scanned using a GeneChip Scanner 3000 7G. Array signal intensities were analyzed with the Affymetrix tools. Analysis data background correction and normalization were performed using the affycoretools R library. To define the differential expression profile between vehicle and metformin treatments, the edgeR library was used. Due to the number of samples per condition being only three, LogFold change (LFC) was set between ≥0.75  and ≤ -0.75. Those *p* values < 0.01 were considered as statistically significant.

### Over-representation analysis

Gene over-representation analysis allows us to observe gene-sets that might participate in a coordinated manner in a determined biological process for a specific phenotype [26]. We decided to implement an over-representation analysis using separately overexpressed and underexpressed genes.

For over-representation analysis, GProfileR online tool [27] was used. Significant values were set to *p* < 0.01. The bubble plot was conducted in R software version 3.5.2 (https://cran.r-project.org; https://www.bioconductor.org.ggplots). Biological pathways reported are those enriched with a *p* < 0.05 significant activation score.

### Quantitative reverse transcription PCR (RT-qPCR) assays

To validate microarray results, the following genes EGR1, GPR183, NR4A1, FOSB, ZFP36, COL1A2, and NR4A3 were selected for RT-qPCR. cDNA was generated using 400 μg of RNA (tumor tissue) with the RevertAid First Strand cDNA Synthesis Kit (ThermoFisher Scientific, USA, K1622) following the manufacturer’s protocol from three different mice treated either with water or with metformin, both treatments were performed at 24 days. Samples employed for RT-qPCR included those used for gene microarray assays and samples from other mice that were part of the original experiment.

qPCR was performed using a 2X Maxima SYBR Green qPCR master mix (Thermo Fisher Scientific, USA) and the Rotor-Gene Q equipment (QIAGEN, Germany). All reactions were run in triplicate. Gene expression fold change was determined with the 2^−ΔCT^ method [28] using GAPDH as the housekeeping gene. Primers used for RT-qPCR are shown in Table 1.

**Table 1:** RT-qPCR primers

Gene	Forward (5´ to 3´)	Reverse (5´ to 3´)
EGR1	GGTCAGTGGCCTAGTGAGC	GTGCCGCTGAGTAAATGGGA
GPR183	GGGAAACTTACTAGCCTTGGTC	GGGAAACTTACTAGCCTTGGTC
NR4A1	CTCTGGAGGTCATCCGCAAG	CTGGCTTAGACCTGTACGCC
FOSB	GCTGCAAGATCCCCTACGAAG	ACGAAGAAGTGTACGAAGGGTT
ZFP36	GACTGAGCTATGTCGGACCTT	GAGTTCCGTCTTGTATTTGGGG
ATF3	ACGGAGTGCCTGCAGAAAG	TCTCGTTCTTGAGCTCCTCA
NR4A3	ATAGTCTGAAAGGGAGGAGAGGTC	TCTGGGTGTTGAGTCTGTTAAAGC
GAPDH	TCCTGCACCACCAACTGCTTA	CATCACTCCACAGYTTYCCAGAG

### Database analysis

TNMplot [29] was employed to determine the expression of EGR1, FOSB, GPR183, ATF3, NR4A1, and ZFP36 in both, normal cervical tissue, and cervical cancer tumors tissue. For ZFP36 gene correlation analysis, Gene Expression Profiling Interactive Analysis (GEPIA) was employed using data from Cervical Squamous Cell Carcinoma and Endocervical Adenocarcinoma considering the Spearman´s coefficient [30].

### Statistical analysis

Data were analyzed using GraphPad Prism (v6; GraphPad Software, Inc, CA, USA). The comparison between two groups was analyzed by unpaired Student’s t-test or Mann–Whitney U test, while multiple groups were analyzed by ANOVA followed by Bonferroni´s or Dunnett´s post-hoc test used when appropriated. Values with a *p* < 0.05 were considered as statistically significant.

## Results

### Metformin reduces CC-derived tumor growth and induces changes in gene expression

**Fig. 1:**
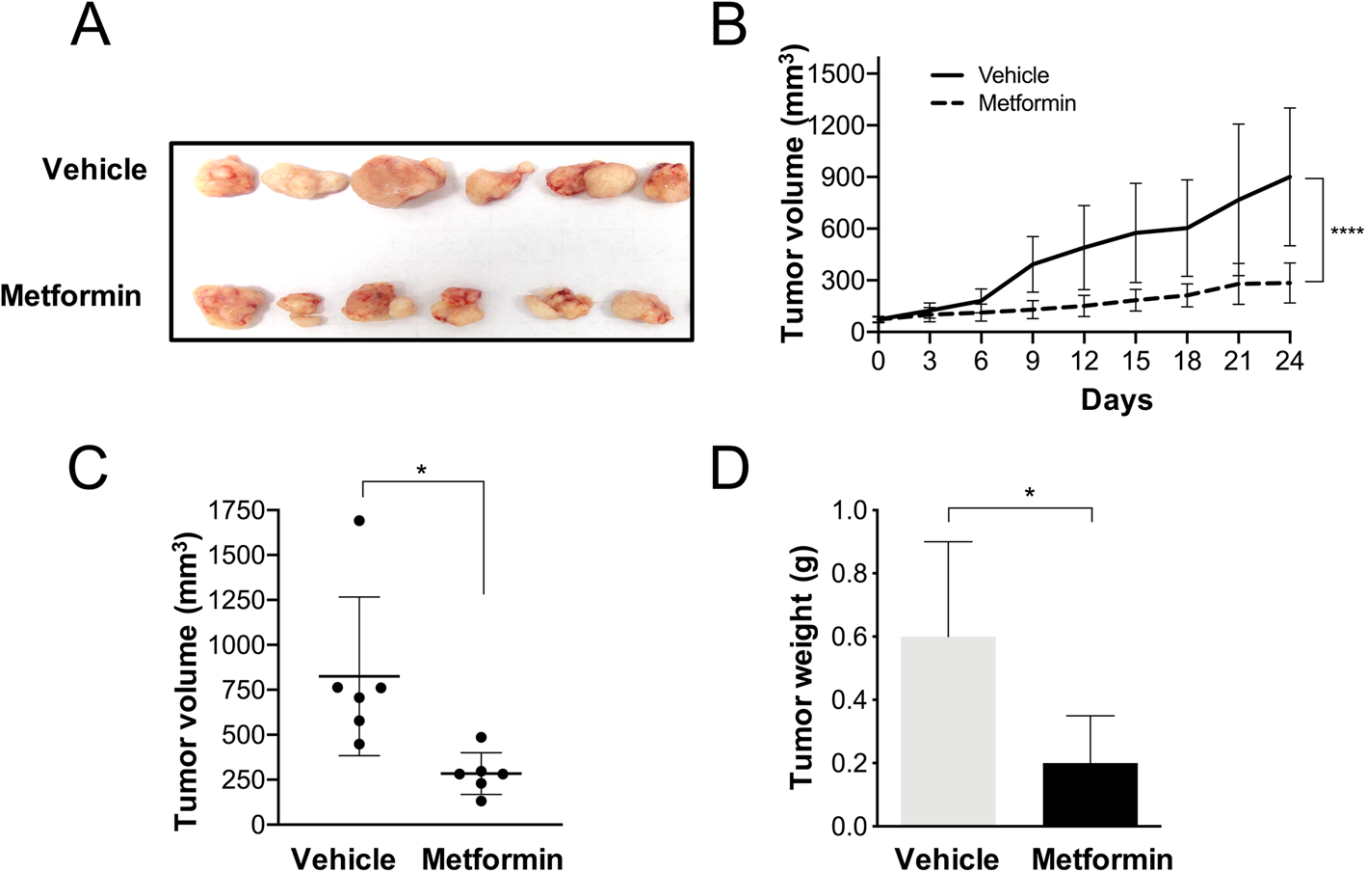
Metformin reduces tumor growth in NOD-SCID mice. Metformin was administered for 24 days as described in the Material and Methods section. **A** Pictured are vehicle (top row) and metformin-treated (bottom row) xenograft tumors. **B** Tumor growth curve (**C**) tumor volume and (**D**) tumor weight of mice treated with metformin by 24 days. Data expressed as mean + DS. *p <0.05, **** *p*<0.001

To investigate the metformin effect on tumor growth, CC-derived xenografts tumors were used. These tumors were generated by subcutaneously injecting HeLa cell suspensions into NOD-SCID mice. It was found that daily metformin treatment significantly reduced tumor growth (Fig. 1A-B). Mean tumor volume was 284.72 mm^3^ in the metformin-treated group, compared with 900.24 mm^3^ from the vehicle-treated group representing a reduction of 68% (Fig. 1B). Consistently, mean tumor weight was statistically lower in metformin-treated group (0.2 g) than the mean tumor weight from vehicle-treated mice (0.6 g) (*p* < 0.05) (Fig. 1D). Importantly, metformin treatment does not produce any toxic effects as indicated by the mice weight after treatments compared to those vehicle-treated mice (data not shown). These results confirm that oral metformin treatment on NOD-SCID mice reduces tumor xenograft growth of CC-derived cells.

**Fig. 2:**
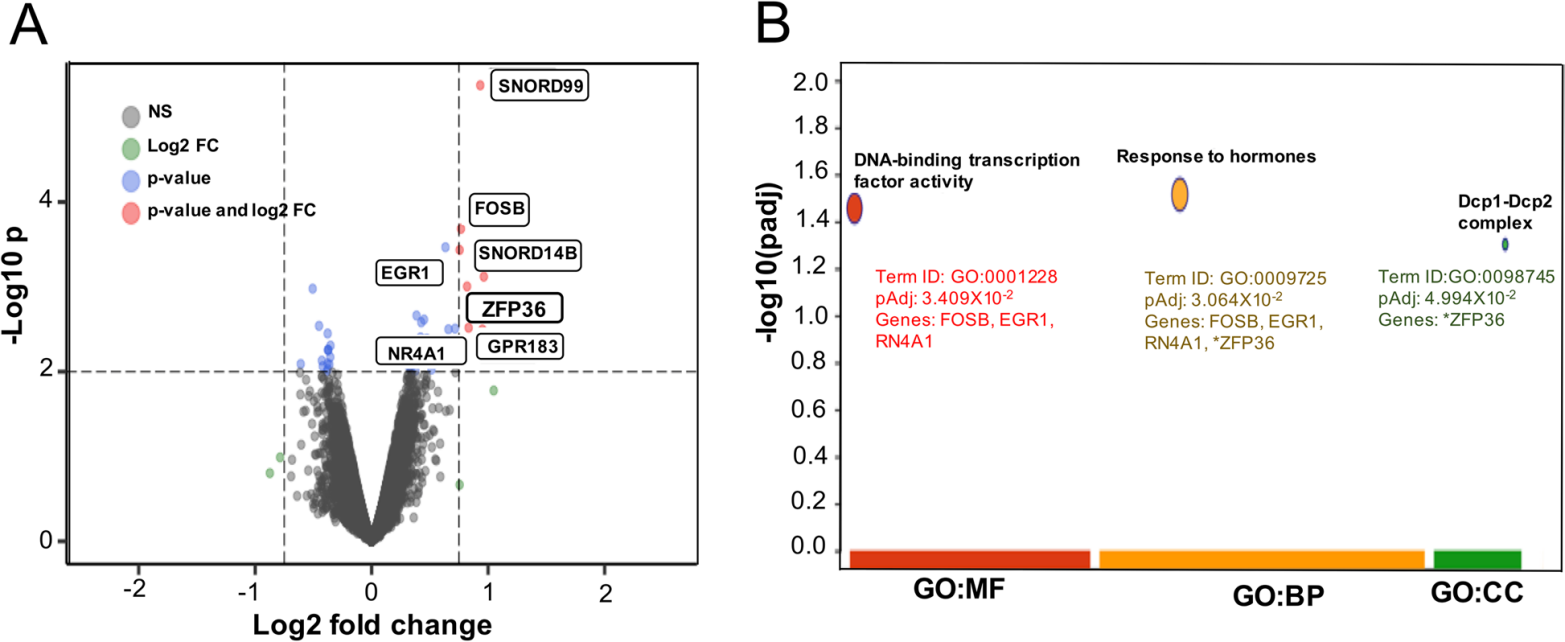
Metformin reduces tumor growth *in vivo* by modulating differently gene expression associated with the regulation of transcription, response to hormones, and mRNA stability. **A** Volcano plot shows genes upregulated in tumors from mice in metformin treatment. Red dots represent differentially expressed non-coding RNAs and genes at significant values (LFC ≤-0.75 and ≥0.75 and *p*<0.01). Functional enrichment analysis of upregulated differentially expressed genes (DEGs) between vehicle and metformin treatment group tumors. **B** Results of enrichment analysis are presented in the form of a Bubble plot, where the x-axis shows the functional terms grouped by the color code of the source database used, while the y-axis shows the enrichment adjusted *p*-values in negative decimal logarithm scale. Size of the bubble represents the score of each pathway. Metformin induces differential expression of genes associated with DNA-binding transcription factor activity, response to hormones, and Dcp1-Dcp2 complex MF: Molecular Function; BP: Biological process; CC: Cellular component

To dissect a transcriptional profile associated to metformin during tumoral inhibition, gene expression profiles employing RNA microarray was analyzed in HeLa cells tumor xenografts from mice treated with metformin for 24 days comparing to the vehicle-treated control. As expected, a differential gene expression profile was observed using a Log fold change (LFC) above 0.75 or under -0.75 and *p*-values lower than 0.01. A total of seven genes were found to be differentially expressed and upregulated by metformin treatment (Fig. 2A). Among them, FOSB, EGR1, ZFP36, NR4A1, and GPR183, as well as two small nucleolar RNAs (SNORD99 and SNORD14B) were found. These genes are known to be involved in transcriptional regulation and exhibit tumor-specific pro-oncogenic or tumor suppressor-like activities in several cancers [31, 32].

To gain insights into cellular processes affected by metformin treatment, a gene over-representation analysis was performed. We found that processes associated to overexpressed genes following metformin treatment include DNA-binding transcription activator activity, hormonal response, and Dcp1-Dcp2 mRNA-decapping complex. Regarding hormonal response, significantly enriched genes were: FOSB, EGR1, ZFP36, and NR4A1 (Fig. 1B).

We further validate changes identified in the transcriptional profile, hence a group of genes clinically relevant that were upregulated with metformin treatment showing a *p*-value < 0.01 and with an LFC higher than 0.75 were selected. Expression levels of this gene group which included ZFP36, NR4A1, EGR1, FOSB, and GPR183, was assessed by quantitative RT-qPCR experiments (Supplementary Fig. 1A-H). Expression levels of another gene group that were upregulated by metformin with a *p*-value < 0.01 and an LFC lower than 0.75 but higher than 0.6 was also quantified. This group included ATF3 and NR4A3. Consistent with our microarray data, it was found increased mRNA levels of all genes analyzed (ZFP36, NR4A1, EGR1, FOSB, GPR183, ATF3, and NR4A3) in the tumors of mice treated with metformin (Supplementary Fig. 1A-H). These results confirm that expression of these genes was significantly higher (*p* < 0.05) in tumors from mice treated with metformin compared to those tumors from the vehicle (Supplementary Fig. 1A-H). Importantly, a greater increase in ZFP36 gene expression in tumors treated with metformin was observed (Supplementary Fig. 1A).

### Expression of ZFP36 is significantly lower in human CC than in normal tissue

To further investigate their relevance and possible role in human cancer, expression of genes that were upregulated by metformin in HeLa cell xenografts were evaluated in an in silico transcripts analysis from patients with CC compared with non-tumoral samples. Expression of ZFP36, NR4A1, EGR1, FOSB, ATF3, and GPR183 was analyzed using the TNM plot database [33]. Interestingly, it was found that these genes were specifically and differentially under-expressed in CC biopsies compared with non-tumoral samples (*p* < 0.01) (Supplementary Fig. 2A-F).

**Fig. 3:**
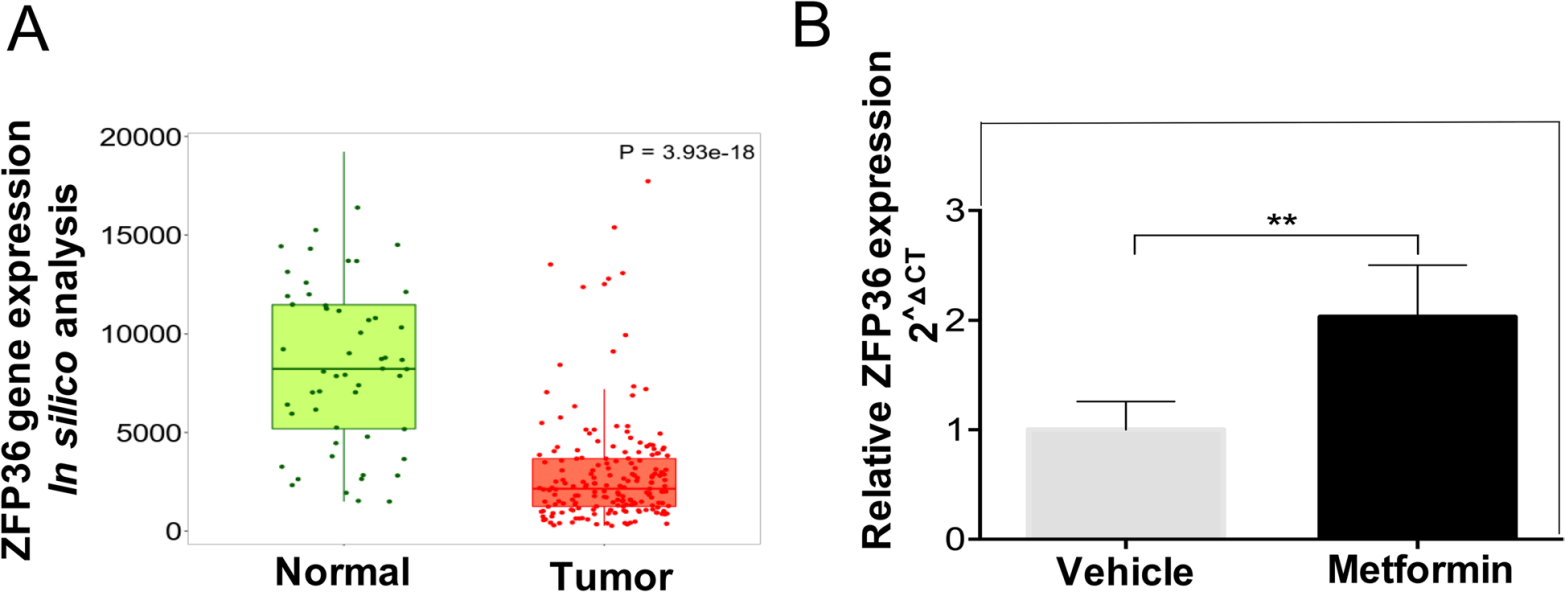
ZFP36 is under-expressed in cervical cancer and metformin increases its expression in xenograft tumors. **A** Boxplot of the expression values from ZFP36 in cervical cancer and normal tissues. Data was obtained from Gene chip data at TNMplot.com. **B** Metformin treatment for 24 days induces an increased expression of ZFP36. RNA from tumors from NOD-SCID mice was used to perform RT-qPCR analysis. Relative amounts of each gene were determined using the Ct method, normalized to GAPDH. Results are shown as means ± SD of at least three independent experiments. *p<0.05 **, *p*<0.01

Due to higher levels of ZPF36 induced by metformin were detected in xenografts, and the cumulative evidence indicating its role in tumorigenesis and metabolic regulation in CC [34–37], we aimed to evaluate ZPF36 levels in CC compared to normal tissue. As shown in Fig. 3A, ZFP36 expression is decreased in tumor tissue from CC patients. Moreover, we demonstrate that metformin treatment induces the ZFP36 expression in HeLa cell xenografts (Fig. 3B) proposing ZFP36 as a transcriptional target for metformin.

To further explore the role of ZFP36 in CC, GEPIA database was employed to measure the co-relationship between the mRNAs that were overexpressed with metformin, NR4A1, EGR1, FOSB, ATF3, and GPR183 in CC patients [33]. It was observed that ZFP36 transcript showed a positive correlation to other transcripts analyzed including NR4A1, EGR1, FOSB, ATF3 and GPR183 (Supplementary Fig. 2G-K). Moreover, these transcripts expression pattern in cancer patients (tumoral samples) revealed a significantly decreased expression compared to non-tumoral samples (Supplementary Fig. 2A-F). These evidences suggest that metformin induces the expression of genes NR4A1, EGR1, FOSB, ATF3 and GPR183 that are underexpressed in CC. Furthermore, it is suggested that ZFP36 could be regulating the expression of these genes.

### Metformin suppresses mTORC1 pathway and induces ZFP36 in tumor xenografts

**Fig. 4:**
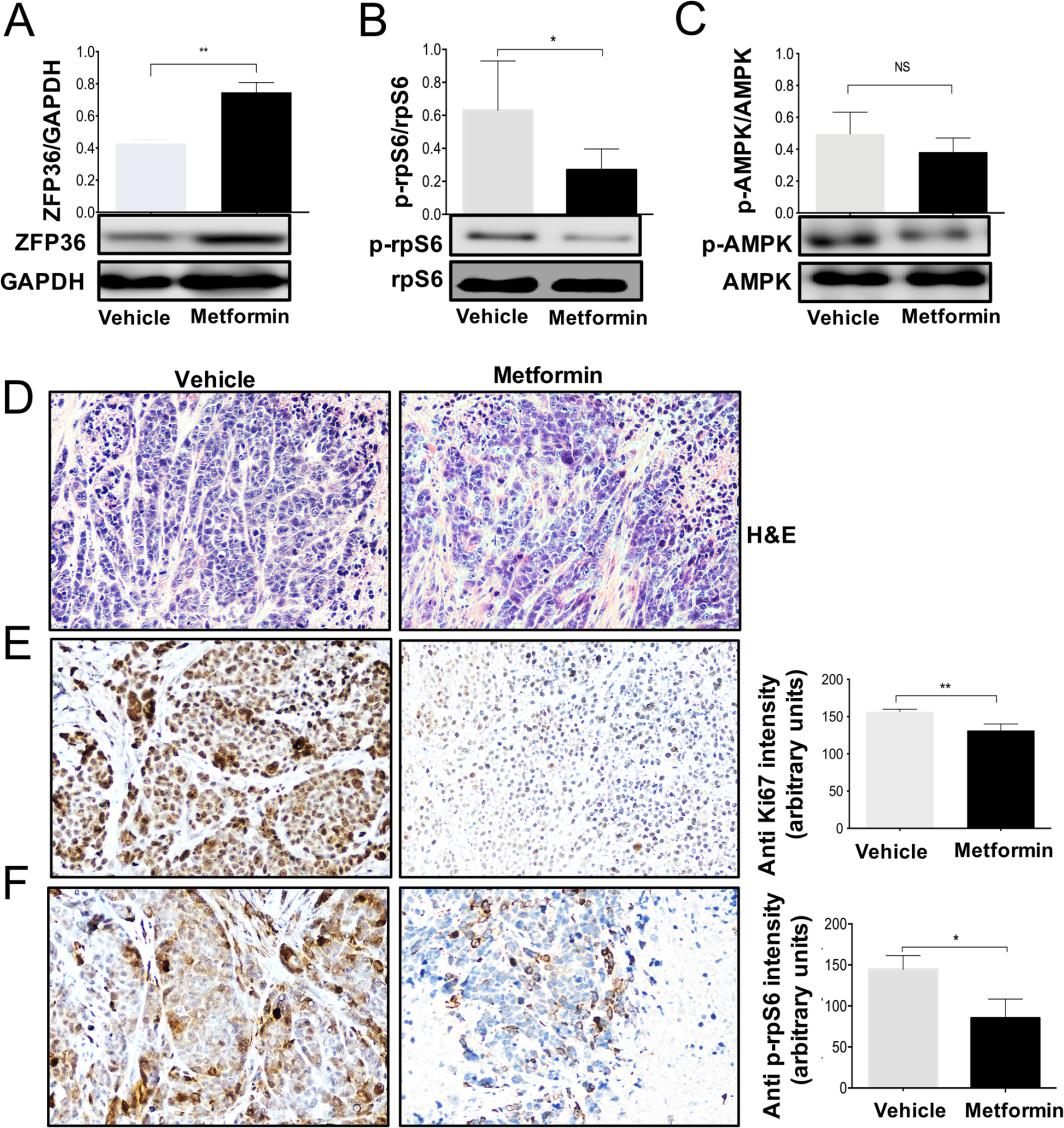
Metformin increases ZFP36 expression by downregulating mTORC1 *in vivo.*Western Blot and densitometric analysis of (**A**) ZFP36, (**B**) p-rps6 (**B**) and (**C**) p-AMPK in tumors from vehicle or metformin treated mice. **D** H&E and (**E** and **F**) IHCs against Ki67 and p-rpS6 of tumor sections from mice treated with metformin or vehicle. Semi-quantitative analysis of Ki67- and p-rpS6-positive cells. Data expressed as mean ± SD of three different mice. * *p* <0.05. 20X magnification

Expression analysis by microarrays indicate that metformin could induce the expression of the ZFP36. Then, we decided to evaluate if metformin also could induce the protein levels of ZFP36 in HeLa xenograft tumors. It was found that metformin treatment induces increased levels of the ZFP36 protein in all xenograft tumors (Fig. 4A). It has been shown that one of the main mechanisms of action of metformin is through the AMPK-mTORC1 axis regulation [1]. For this reason, we were interested in evaluating whether metformin acts through these targets. To assess whether metformin inhibits mTORC1, the phosphorylation of the rpS6 protein (p-rpS6) was evaluated by Western Blot. In the case of the metformin group, a decreased phosphorylation state of rpS6 was found (*p* < 0.05) (Fig. 4B). However, p-AMPK does not show any effect by metformin treatment concerning the vehicle group (Fig. 4C).

Histopathological changes associated with metformin treatment were assessed using paraffin sections of tumors from each group by H&E staining. In Fig. 4D, a low cellularity was observed in sections from metformin-treated tumors in comparison with those from the vehicle group. Next, to examine whether metformin decreased the tumor volume of HeLa xenografts by down-regulating cell proliferation, an in situ Ki67 staining was performed. Ki67 IHC staining quantification indicated that tumors from metformin treatment mice showed significantly less Ki67-positive cells (*p* < 0.05) as compared to tumors from mice treated with vehicle (Fig. 4E). Moreover, IHC assays showed that p-rpS6 was significantly reduced in tumor cells from mice treated with metformin for 24 days (*p* < 0.05) (Fig. 4F). These results indicated that metformin probably inhibits tumor growth in vivo by decreasing cellular proliferation rates through mTORC1 inhibition and ZFP36 induction.

### Metformin induces ZFP36 expression in CC-derived cell lines but not in non-tumor cells

**Fig. 5:**
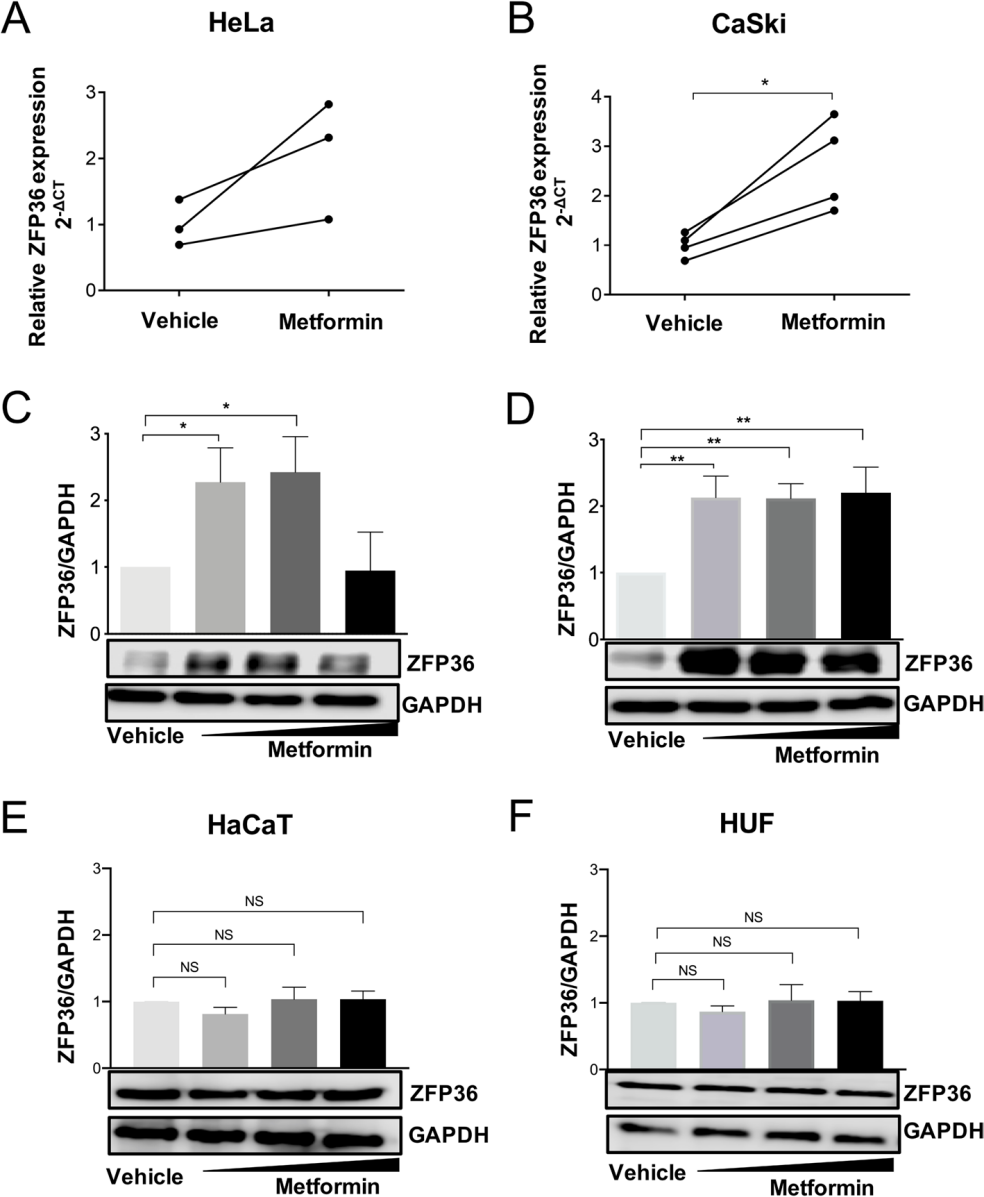
Metformin induces ZFP36 expression in cervical cancer-derived-cell lines. HeLa and CaSki cells were treated with metformin 20mM for 24 hours, then RNA was extracted for analysis by RT-qPCR. RT-qPCR of ZFP36 after metformin treatment in (**A**) HeLa and (**B**) CaSki. Representative Western Blot of ZFP36 and densitometric analysis in (**C**) HeLa and (**D**) CaSki, (**E**) HaCaT and (**F**) Human Uterine Fibroblast (HUF). Results are shown as means ± SD of at least three independent experiments. *p<0.05 **, *p*<0.01

The next step was to determine whether metformin induces ZFP36 expression in CC-derived cell lines. For this purpose, HPV-positive CC-derived cell lines HeLa and CaSki were employed. First, the ZFP36 relative expression was measured by RT-qPCR in both cancer cell lines using a treatment of 20 mM of metformin for 24 h. Results indicated a significant increase in ZFP36 expression induced by metformin compared to the vehicle in HeLa and CaSki cell lines (Fig. 5A, B). Further, Western Blot analysis was performed to validate if, similarly to its mRNA, ZFP36 protein can also be increased. Using 5 mM, 10 mM, and 20 mM of metformin in HeLa and CaSki cells, an increase of ZFP36 protein levels was observed. In the case of HeLa cells, this effect seems to be at lower metformin concentrations, while in CaSki cells the effect was observed in all doses used (Fig. 5C, D). To demonstrate that the induction of ZFP36 by metformin is specific to CC cells, the HaCaT cell line was used as well as a primary culture of Human Uterine Fibroblast (HUF). Using 5 mM, 10 mM, and 20 mM metformin for 24 h, results indicated no changes in the ZFP36 protein induced by metformin in non-tumor cells. These findings suggest that metformin treatment could be directly inducing ZFP36 expression in vivo and in vitro in CC but not in non-tumor cells.

### ZFP36 expression induced by metformin requires mTORC1 pathway down-regulation

To define the mechanism that enables ZFP36 expression by metformin, the potential processes behind this pathway were explored. As mentioned before, contrasting evidence sustains that AMPK/mTORC1 axis acts in a biphasic way to modulate the tumor suppressor effect exerted by metformin [38–40]. Hence, the P70S6K1 and rpS6 (two downstream effectors of the mTORC1 pathway) phosphorylation status was measured as means of mTORC1 activity [41–43].

**Fig. 6:**
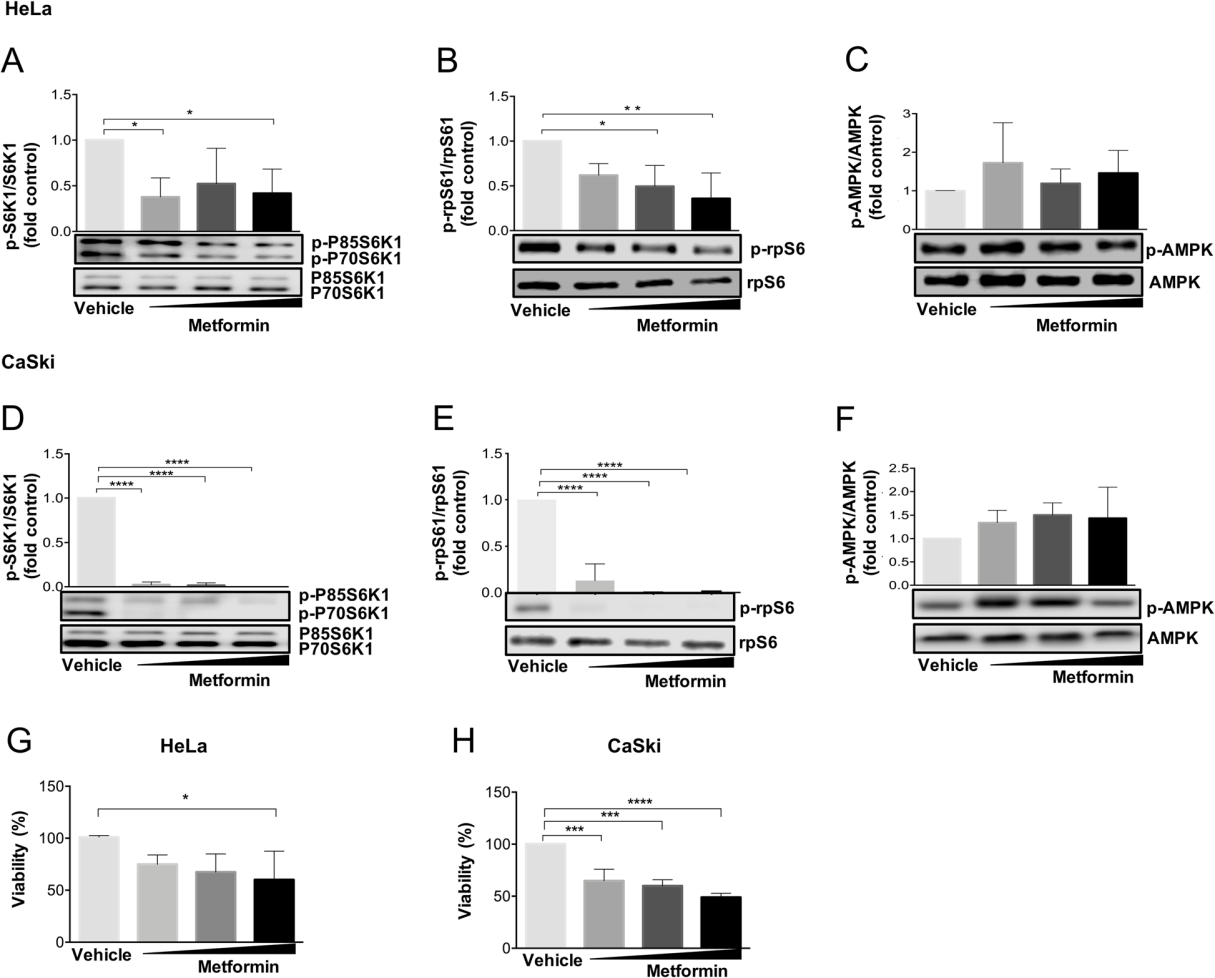
Metformin downregulates mTORC1/S6K1/rpS6 pathway and decreases cell viability in cervical cancer cell lines. HeLa and CaSki cells were treated with different metformin concentrations for 24 hours and, the levels of p-P70/P85 S6K1, p-rpS6, and p-AMPK were measured by Western Blot in HeLa cells (**A**-**C**) and CaSki cells (**D**-**F**). **E** and **F** HeLa cells and CaSki cells were treated with 5, 10, and 20 mM of metformin for 24 h. Then, cell viability was examined using an MTT assay. The plots represent the densitometric and statistical analysis of Western Blot and data expressed as mean + SD of at least three independent experiments. *p< 0.05, **p< 0.01, ****p< 0.001

Protein extracts from HeLa and CaSki cells treated with 5 mM, 10 mM, and 20 mM of metformin for 24 h were used to evaluate the phosphorylation of P70S6K1 at Thr389 by Western Blot analysis. Results revealed that metformin treatment reduced the phosphorylation of P70S6K1 in both cell lines, the responsiveness seems to be differential. While metformin significantly suppressed P70S6K1 phosphorylation in HeLa cells in a dose-dependent manner (Fig. 6A). In CaSki cells, a substantial suppression of P70S6K1 phosphorylation, even at low doses of metformin was observed (Fig. 6D). Moreover, it was found that metformin also decreases phosphorylation of S6K1 isoform known as P85S6K1 which is also a target of mTORC1 (Fig. 6A and D). To corroborate if, the P70S6K1 decreased phosphorylation reflects changes in its activation, the phosphorylation status of rpS6 at Ser235/236, a downstream target of P70S6K1, was examined. Consistently, as shown in Fig. 6B and 6E, metformin significantly decreased rpS6 phosphorylation in HeLa and CaSki cell lines at the indicated doses (*p* < 0.05). Although, it has been shown that AMPK activity could be decreasing mTORC1 activity, AMPK phosphorylation exhibited only a slightly but not significant increase when cells were treated with metformin (Fig. 6C, F). Since we observed that metformin inhibits mTORC1 differently in these cell lines, we decided to evaluate the effect of metformin on cell viability. Then, the effect of different metformin concentrations (5 mM, 10 mM, and 20 mM) on the cellular viability of HeLa and CaSki cell lines treated for 24 h was measured using mitochondrial reduction of MTT. Results showed that treatment with 20 mM metformin significantly decreased HeLa cell viability (*p* < 0.05) (Fig. 6G). Interestingly, CaSki cells were more sensitive to metformin in a dose-dependent manner since a lower concentration (5 mM) significantly reduced cell viability (*p* < 0.05) (Fig. 6H).

**Fig. 7:**
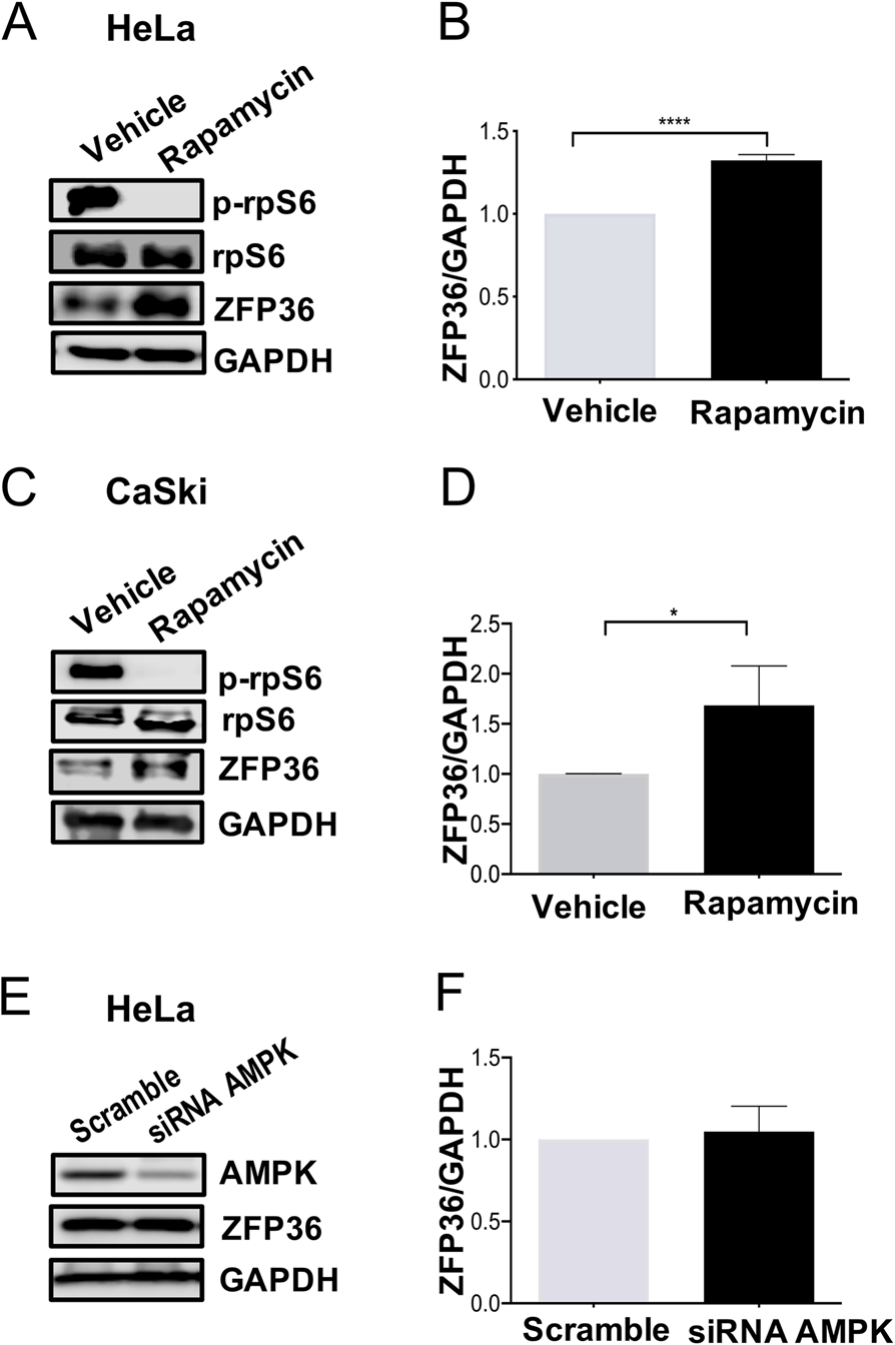
ZFP36 is induced by mTORC1 inhibition in CC cells. HeLa and CaSki cells were treated with 50 nM rapamycin or with vehicle (ethanol) for 24 h, and the levels of ZFP36 and p-rpS6 were measured by Western Blot. **A** and **B** Corresponding Western Blot and densitometric analysis of ZFP36/GAPDH in HeLa cells. **C** and **D** Corresponding Western Blot and densitometric analysis of ZFP36/GAPDH in CaSki cells. **E** HeLa cells were transfected with control or AMPK-targeting siRNAs for 24 h, and the levels of AMPK and ZFP36 were measured by Western Blot. **F** Corresponding densitometric analysis of ZFP36/GAPDH. The plots represent the densitometric and statistical analysis of Western Blot and data expressed as mean + SD of at least three independent experiments. *p< 0.05, **p< 0.01

Although the above results point out ZFP36 expression is induced by metformin, the dependence of mTORC1 or AMPK axis for such a process needs to be addressed. Hence, we further explored the direct inhibition effect of mTORC1 over ZFP36 expression. By employing rapamycin, a well-known allosteric inhibitor of mTORC1, at 50 nM for 24 h we measure it influence on ZFP36 protein levels in HeLa and CaSki cells. As expected, rapamycin decreases the downstream effector p-rpS6, validating its mechanistic functions over the mTORC1 pathway (Fig. 7A-D). Interestingly, this effect leads to a significant increase in ZFP36 levels both in HeLa and CaSki (Fig. 7C, D). Since mTORC1 activity is intrinsically associated with AMPK activity, it was tested in HeLa cells if AMPK could be modulating the effect over ZFP36. Therefore, AMPKα1 and AMPKα2 subunits were knock-down using AMPK-targeted siRNAs achieving a significant AMPK subunits knockdown at 24 h and up to 48 h post-transfection (data not shown). Interestingly, our results reveal non-changes in ZFP36 expression by knocking down AMPK in HeLa cells, confirming that mTORC1 down-regulation could be necessary to induce ZFP36 protein levels (Fig. 7E-F), whereas AMPK could be dispensable for such effect.

**Fig. 8:**
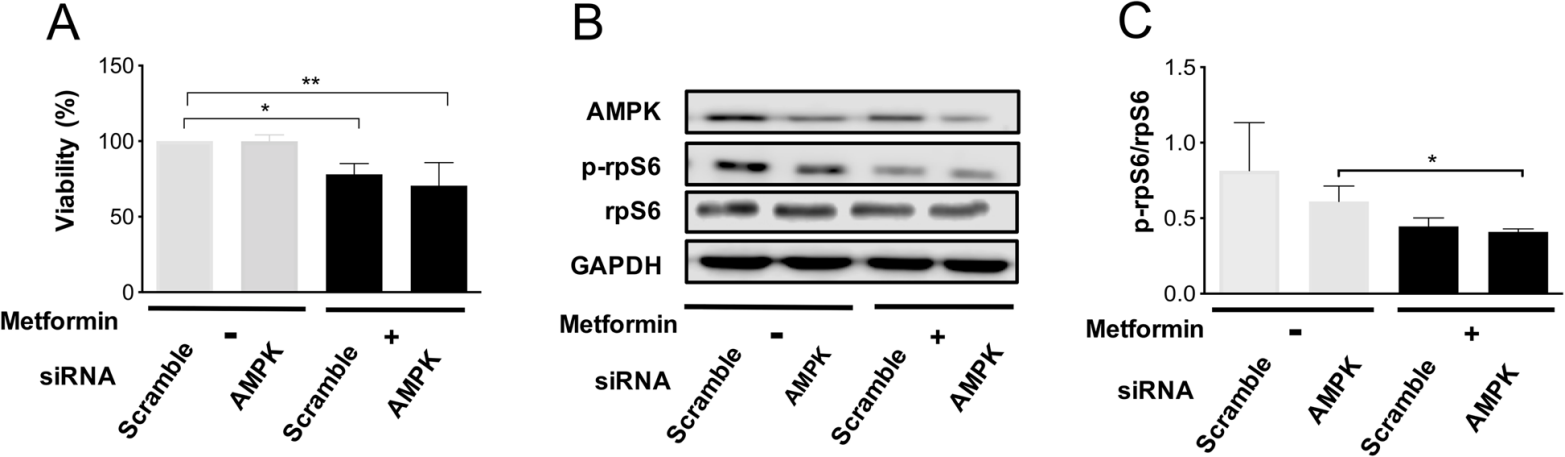
Metformin inhibits mTORC1 and cell viability independently of AMPK. HeLa cells were transfected with control or AMPK-targeting siRNAs and treated or not with 20 mM metformin for 24 h. **A** Cell viability was examined using an MTT assay. **B** The levels of AMPK, p-rpS6, rpS6 were measured by Western Blot. **C** Corresponding densitometric analysis of p-rpS6/rpS6. Data expressed as mean + SD of at least three independent experiments. *p< 0.05, **p< 0.01

It was observed that AMPK is not involved ZFP36 expression induced by metformin in CC. Hence, we decided to evaluate whether AMPK is participating in the decrease of cell viability and the regulation of mTORC1 induced by metformin. To this end, expression of AMPK was inhibited using siRNAs and examined the effects of AMPK knock-down on the antiproliferative activity of metformin in HeLa cells. It was observed that either in Scramble or AMPK knockdown, metformin decreases cell viability, thus confirming that the metformin effect could be independent of AMPK (*p* < 0.05) (Fig. 8A and B). Next, it was tested whether the inhibition of mTORC1 induced by metformin is dependent of AMPK in HeLa cells. Hence, AMPK expression was inhibited using siRNA and the effects on the rpS6 phosphorylation were evaluated after metformin treatment (20 mM) during 24 h. Surprisingly, it was observed that metformin could not possibly be acting through AMPK for mTORC1 inhibition since it was shown that AMPK knockdown did not affect the rpS6 phosphorylation in Hela cells (Fig. 8 B and C).

Input and output signals might control mTORC1 activity [44, 45]. mTORC1 activity is regulated through phosphorylation by multiple upstream kinases [46]. For instance, it has been widely reported that the Erk1/2 pathway enables the activity of mTORC1 [47]. For this reason, it was decided to evaluate whether metformin treatment alters the Erk1/2 phosphorylation status. Treatment of CC-derived cells with metformin for 24 h significantly decreased Erk1/2 phosphorylation at Tyr204. While in HeLa cells a significant reduction was observed with 5 mM and 20 mM of metformin (Supplementary Fig. 3A). CaSki cells showed a dose-dependent inhibition and a significant reduction at 10 mM; and 20 mM (Supplementary Fig. 3B). These results suggest that metformin could be decreasing the mTORC1/S6K1/rpS6 pathway, at least by Erk1/2 signaling downregulation.

With these results, it was verified that metformin inhibits mTORC1 whereas not involve AMPK activity and suggest that, at least in a CC model, metformin decreases the growth of HeLa xenografts in NOD-SCID mice through the induction of ZFP36 via mTORC1 inhibition.

## Discussion

Metformin has been shown to exhibit different antitumoral effects, including a reduced gynecological cancer rate as well as an increased response to treatment in breast and lung cancer patients [32]. One of the most accepted mechanisms by which metformin acts is by inhibiting the mitochondrial respiratory complex I and activating AMPK in response to energy depletion [8]. However, other signaling pathways might contribute to the metformin antitumoral effects. Studies in CC have demonstrated that metformin reduces cellular growth by targeting the Wnt/DVL3 pathway [33], or by modulating the expression of FOXM1 [34]. Moreover, metformin has also been shown to inhibit CC cells migration and invasion by decreasing the long non-coding RNA MALAT1 expression [35] and by regulating protein expression associated with the insulin signaling pathway [36]. Although, several studies have posed the antitumoral effects of metformin in CC, alternative mechanisms are not entirely understood.

In this study, we analyzed changes in the transcriptomic profile of tumors xenografted with HeLa cells in mice treated with metformin. It was demonstrated that metformin induces ZFP36 expression through the possible mTORC1 inhibition in an AMPK-independent manner, in vivo and in vitro assays. Specifically, it was found that metformin increased the expression of a group of antitumoral genes, particularly the ZFP36 expression which was significantly induced by metformin. Research has shown that ZFP36 regulates tumorigenesis by destabilizing the expression of critical genes implicated in both, tumor onset and tumor progression, such as HK2, HIF1α, and c-Myc [48]. Strikingly, treatment with metformin might reduce tumor growth, mainly by inducing the expression of a set of genes FOSB, EGR1, ZFP36, GPR183, ATF3, and NR4A1 which are involved in DNA-binding transcriptional activation, response to hormones and the Dcp1-Dcp2 mRNA-decapping complex. Interestingly, most of these genes are enriched in the hormone response process (FOSB, EGR1, ZFP36, and NR4A1). This is consistent with the metformin anti-tumoral effects which are not limited to its direct effects on cancer cells, but also in changes on the systemic level due to metformin may exert antitumor effects by reducing insulin levels [48].

Our *in-silico* data revealed that NR4A1, EGR1, FOSB, GPR183, ATF3, and ZFP36 were down regulated in tissue from patients with CC. Thus, restored expression of this set of transcripts by metformin might be therapeutically feasible. Recently, it was shown that components of the AP-1 transcription factor (FOS, FOSB, JUN), EGR1, and NR4A1 behave as a conserved co-regulated group of genes whose expression is associated with ZFP36 in cancer cells [47]. Low ZFP36 expression levels were associated with poor prognosis in breast cancer patients [47]. Interestingly, we found that genes such as ZFP36, NR4A1, FOSB, and EGR1 were positively regulated by metformin (Supplementary Fig. 1). These results support the idea that ZFP36 gene network could be important for mediating the metformin antitumoral response.

One metformin main mechanisms of action involve mTORC1 inhibition [49]. mTORC1 regulates a wide variety of genes involved in a multitude of tumoral cellular processes, such as protein translation, autophagy, lysosome biogenesis, lipid synthesis, and growth factor signaling [46, 50, 51]. In a study carried out in cardiomyocytes, it was observed that mTORC1 inhibits the expression of ZFP36 [52]. Thus, we hypothesized that the induction of ZFP36 by metformin treatment in CC might be explained by mTORC1 inhibition. To verify this idea, we used rapamycin, a widely mTORC1 inhibitor detecting ZFP36 expression in the absence of metformin suggesting that metformin induces ZFP36 expression through the mTORC1 inhibition. However, more experiments are needed to demonstrate the exact mechanism by which mTORC1 inhibits the ZFP36 expression.

Recently, it was shown that metformin induces ZFP36 expression through AMPK activation in breast cancer cell lines [53]. Nonetheless, we did not observe changes at least in HeLa cells in ZFP36 protein levels when AMPK was inhibited, in contrast to evidence provided by Pandiri et al., (2016) [53]. The activity of AMPK induced by metformin is discrete but non-significant at low doses, so it cannot be entirely discarded its influence to mediate mTORC1 activity. Furthermore, in HeLa cells it was observed that metformin decreases cell viability even when AMPK was inhibited. This rises the suggestion that, alternative pathways are regulating the metformin antitumoral effect at least in a CC model. One possible explanation could be the MAPK/Erk/P90S6K pathway, since metformin is capable to inhibit Erk 1/2 phosphorylation in both HeLa and CaSki cells (Supplementary Fig. 3A and B) and evidence has shown that mTORC1 can be activated by Erk 1/2 [54].

Interestingly, with the results obtained from the in silico analysis, it was shown that ZFP36 is under-expressed in samples from patients with CC, which is quite interesting because it was also shown that metformin increases the ZFP36 expression. Moreover, it was found an increase in ZFP36 expression which was correlated with the impairment of mTORC1 activity, both in vivo and in vitro, suggesting that metformin induces the ZFP36 expression via inhibiting mTORC1 in CC. However, more experiments are necessary to verify if metformin affects not only the expression of ZFP36 but also its activity as a regulator of the stability of mRNAs that contain AREs such as c-Myc, HK2, and HIF1-α [55–58], important genes that could be regulated by metformin.

## Conclusions

Transcriptome profile reveals a set of genes regulated by metformin, some of them with proved antitumoral effect, where ZFP36 expression seems to be mediating such effect. Our results demonstrated that metformin induces ZFP36 expression using mTORC1 inhibition, both in vivo and in vitro*.* Whereas AMPK activity is not the main axis induced by metformin. Although the mechanism for ZFP36 regulation remains unclear, our results suggest that metformin inhibits tumor growth through ZFP36 induction via mTORC1 inhibition. More research is needed to understand the metformin mechanisms associated to ZFP36 regulation, other genes identified in this study, as well as others still to be discovered.

### Supplementary Information

Supplementary Material 1.

## Data Availability

The data that support the findings of this study are available from the corresponding author upon reasonable request.

## Abbreviations

ZFP36: Zinc Finger Protein 36
mTORC1: Mammalian or mechanistic target of rapamycin
AMPK: Adenosine monophosphate-activated protein kinase
Erk: Extracellular regulated kinase
ETC: Electron transport chain
S6K1: Ribosomal protein S6 kinase 1
4E-BP: Eukaryotic initiation factor 4E-binding protein
rpS6: Ribosomal protein S6
P90S6K: P90 ribosomal protein S6 kinase
MAPK: Mitogen activated protein kinase
NOD-SCID: Non-obese diabetic-severe combined immunodeficiency
cDNA: Complementary DNA
RT-qPCR: Quantitative reverse transcription PCR
