# Supplementary material for: Metformin induces ZFP36 by mTORC1 inhibition in cervical cancer-derived cell lines
Source: BMC Cancer. 2024 Jul 18;24:853. doi: 10.1186/s12885-024-12555-5 (PMC11256429; doi:10.1186/s12885-024-12555-5)
Supplement: Supplementary file 1 — Supplementary Material 1. [file 12885_2024_12555_MOESM1_ESM.docx]

**ADDITIONAL FILE**

De la Cruz-López et al.

Metformin induces ZFP36 by mTORC1 inhibition in cervical cancer-derived cell lines.

Supplementary Figure 1.

Validation of induced expression of selected genes from tumors of mice treated orally with metformin.

Supplementary Figure 2.

Expression of ZFP36, NR4A1, EGR1, GPR183, FOSB, and ATF3 are significantly lower in human cervical cancer compared to normal tissue.

Supplementary Figure 3.

Metformin decreases Erk 1/2 phosphorylation in cervical cancer cell lines.

**
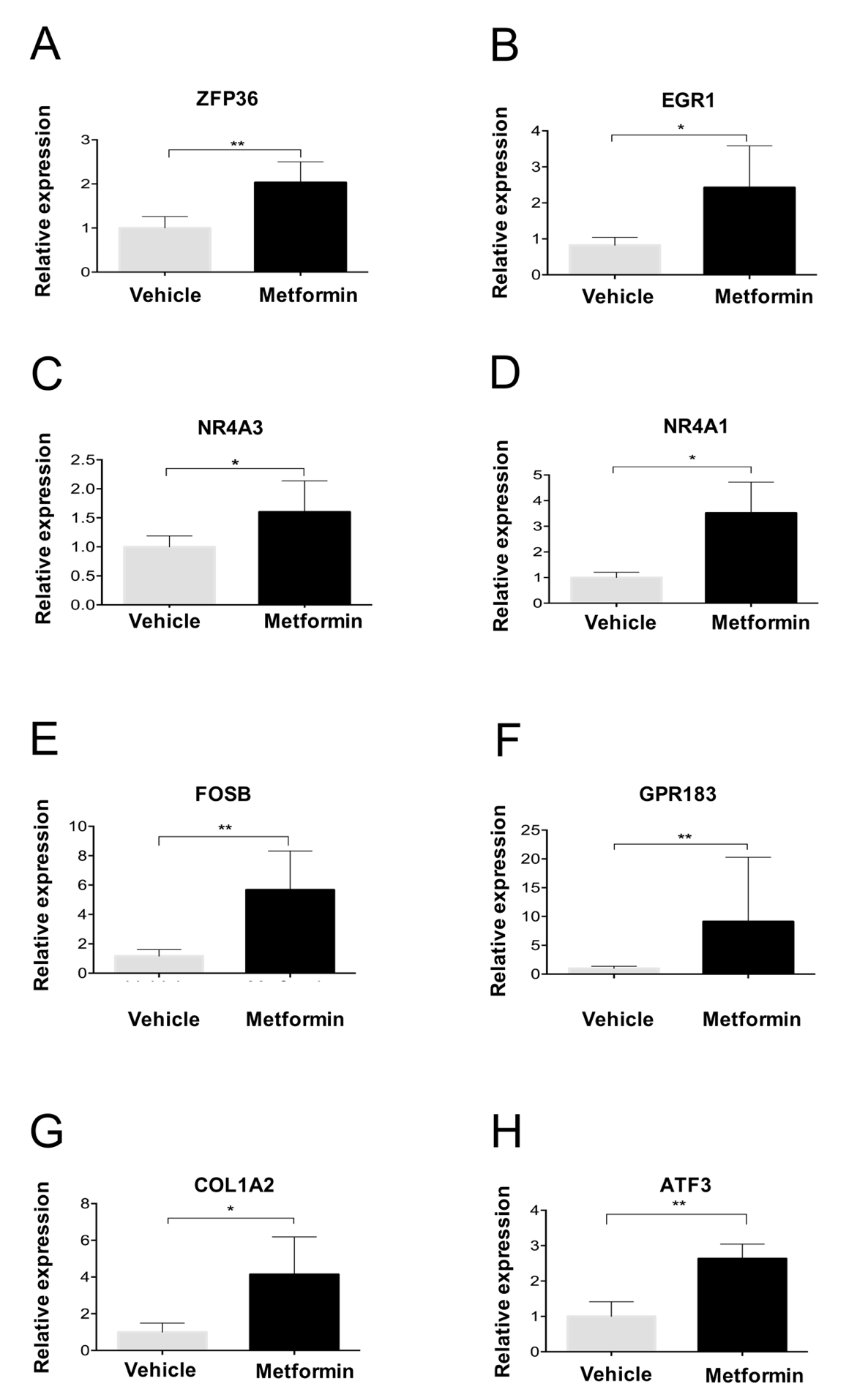
**

**Supplementary Figure 1.** Validation of induced expression of selected genes from tumors of mice treated orally with metformin. As described in Materials and Methods, RNA from tumors was used to perform RT-qPCR analysis. Relative amounts of each gene were determined using the Ct method, normalized to GAPDH. (A-H) Metformin treatment for 24 days induces an increased expression of ZFP36, NR4A1, FOSB, GPR183, NR4A3, EGR1, and ATF3 genes. Data expressed as mean +SD from at least three different mice per group. *p<0.01

**
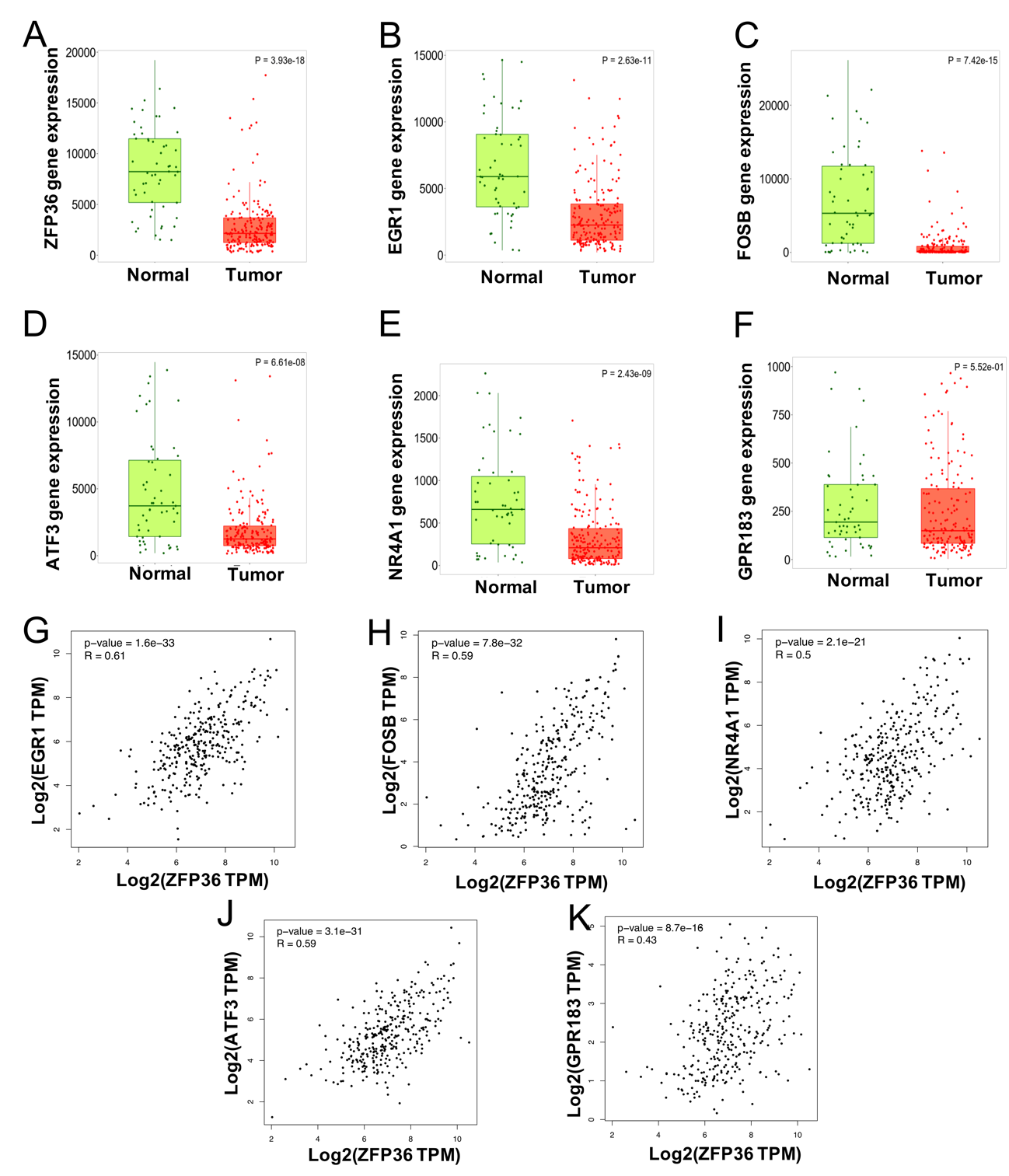
**

**Supplementary Figure 2.** Expression of ZFP36, NR4A1, EGR1, GPR183, FOSB, and ATF3 are significantly lower in human cervical cancer compared to normal tissue. (A-F) Boxplots of the expression values from ZFP36, NR4A1, EGR1, FOSB, GPR183, and ATF3 in cervical cancer and normal tissues. Data was obtained from Gene chip data at TNMplot.com. (G-K) Genes regulated by metformin show a positive correlation with the ZFP36 gene in samples from cervical cancer patients. Correlation analysis among ZFP36 and NR4A1, EGR1, GPR183, FOSB, and ATF3 in cervical cancer tissues. The Mann-Whitney U test was used for expression analyses, and Spearman's test was used for correlation analysis.

**
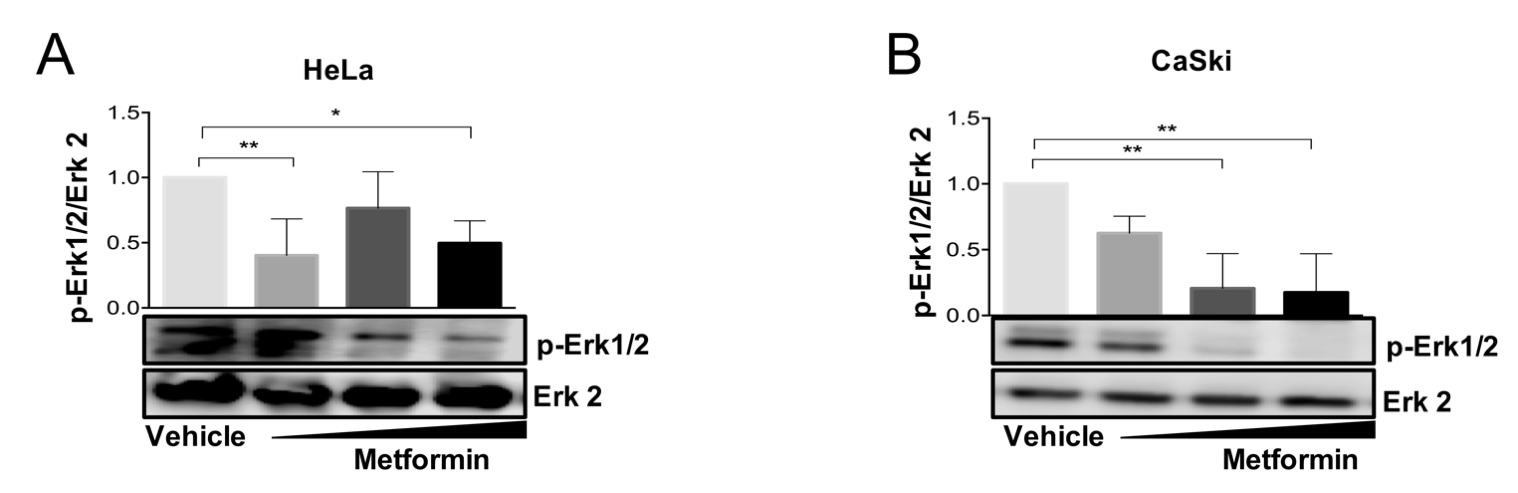
**

**Supplementary Figure 3.** Metformin decreases Erk 1/2 phosphorylation in cervical cancer cell lines. (A) HeLa and (B) CaSki cells were treated with different metformin concentrations for 24 hours and, the levels of p-Erk 1/2 were measured by Western Blot. Results are shown as means ± SD of at least three independent experiments. *p<0.05 **, p<0.01.
